# Associations Between Blast Exposures and Intestinal Permeability and Neurotrauma Symptoms During Mortar Fire Military Tactical Training Operations

**DOI:** 10.1093/milmed/usaf478

**Published:** 2026-05-01

**Authors:** Zhaoyu Wang, Qingkun Liu, Jeffrey Nemes, Alis Kranfli, Molly Sullan, Andrew Hoisington, Lisa A. Brenner, Maciej Skotak, Christina R. LaValle, Yongchao Ge, Walter Carr, Fatemeh Haghighi

**Affiliations:** 1 General Medical Research, James J. Peters VA Medical Center, Bronx, NY 10468, United States; 2 Department of Neuroscience, Icahn School of Medicine at Mount Sinai, New York, NY 10029, United States; 3 Walter Reed Army Institute of Research, Silver Spring, MD 20910, United States; 4 Department of Veterans Affairs, Rocky Mountain Mental Illness, Research, Education and Clinical Care, Aurora, CO 80045, United States; 5 VA Brain Health Coordinating Center, Rocky Mountain Regional VA Medical Center, Aurora, CO 80045, United States; 6 Department of Physical Medicine & Rehabilitation, Anschutz Medical Campus, University of Colorado, Aurora, CO 80045, United States; 7 Department of Systems Engineering and Management, Air Force Institute of Technology, Wright-Patterson Air Force Base, OH 45433, United States

## Abstract

**Introduction:**

Military members are exposed in combat and training to recognized traumatic brain injury (TBI)-causing events and other sub-concussive events that result in physical, psychological, and physiological impacts. Studies by this team and others involving Service Members (SMs) engaged in tactical training operations with repeated exposure to low level blast (LLB) have shown associations with concussion-like symptomology as well as transient decrements in performance, blood-based neurotrauma biomarkers, and perturbations in epigenome and transcriptome profiles, as well as alterations in intestinal permeability (IP). The present study focused on SMs engaged in mortar fire tactical trainings, builds on our previous findings in breachers that identified associations between blast exposures and IP and neurotrauma symptoms following exposures to LLB.

**Materials and Methods:**

Self-report symptom data and blood specimens from 31 SMs were collected including 22 mortarmen who directly participated in tactical training operations and were exposed to LLB and 9 unexposed study controls. Symptom data and blood samples were collected at pre-, post, and follow-up time points, across 3 consecutive mortar fire training sessions. Blood samples were assessed across all sessions via enzyme-linked immunosorbent assay (ELISA) to measure levels of IP protein biomarkers (i.e., Zonulin, Lipopolysaccharide-Binding Protein (LBP), Claudin-3, Intestinal-Fatty Acid Biding Protein (I-FABP). Correlations between IP biomarker changes and magnitude of blast exposures in each training session were calculated and reported as effect sizes measured by Cohen’s d using correlation to effect size conversion via R package effectsize. Impact of blast magnitude on IP biomarker changes across sessions were tested using a linear mixed effect model via R package lme4 and emmeans, with similar models used to investigate associations of IP biomarker changes following blast with physical and psychological symptoms or prior history of TBI.

**Results:**

In mortarmen LLB exposure magnitudes were significantly associated with change in Intestinal-Fatty Acid Binding Protein (pre vs. post, P = .028) and Zonulin (pre vs. follow-up, P = .003) levels across training sessions. Associations with moderate to large effect sizes were observed between changes in LBP and CLDN3 levels and neurotrauma symptoms, including taking longer to think, dizziness, and concentration difficulties following exposures to LLB (Cohen’s ∣d∣ > 0.5). Associations between TBI history and blast induced alterations in LBP and CLDN3 levels were identified with large effect sizes (∣d∣ > 0.8).

**Conclusions:**

The present study in mortarmen corroborates our prior findings showing that exposures to LLB can contribute to IP that is also associated with prior mild traumatic brain injury (mTBI) history, with concomitant decreases in self-reported cognitive functioning. These findings suggest a possible role of blast exposure in gut permeability and the importance of the gut-brain axis in blast injury, with significant clinical translational impact in how we target clinical symptoms associated with exposure to blast and long term sequalae. These results may lead to a paradigm shift in the manner by which the military can detect, mitigate, and treat blast-related sequelae.

## INTRODUCTION

Exposures to improvised explosive devices and other forms of explosive warfare within armed conflicts in Iraq and Afghanistan contributed to elevated rates of traumatic brain injuries (TBI) in our Warfighters and Veterans, with 66% of such injuries in deployment settings having been attributed to blast exposure.^1^ Exposures to blast are not only confined to deployment settings, since Service Members (SMs) are required to engage in military training exercises to maintain military readiness, where they are repeatedly exposed to low-level blasts (LLB). These exercises help service members build familiarity and skill with various weapons systems under realistic conditions; however, repeated exposure to even LLBs can have potential effects on health, particularly on the brain over time. A cross-sectional study of blast overpressure (BOP) exposure for various occupational specialties in operational training environments (indoor and outdoor breaching, shotgun door breaching, small arms discharge, and mortar and artillery fire missions) indicated that the real-world training environmental exposures consistently exceed the tentative 4 psi (28 kPa) incident BOP safety threshold.^2^ Such exposures during breaching and mortar fire tactical training operations are also shown to be associated with reported symptoms of neurotrauma.^3-7^ Although our understanding of the effects and mechanisms of blast injury are limited, investigations of the effects of blast in military tactical training operational settings have begun to address this gap in knowledge.^8,9^

The brain appears to be particularly vulnerable to blast injury.^10^ Blast shockwaves can transmit pressure through the skull where the energy of the shockwave can be absorbed by the brain tissues,^11^ with disruption of the blood brain barrier.^12^ Exposure to blast can also result in profound effects on the gastrointestinal (GI) system through a series of biophysical and pathophysiological mechanisms by the transmission of high-energy shock waves, who interact with the body’s gas-containing organs. When a blast wave strikes the body, a fraction of its energy is reflected, some is deflected, and a substantial portion is transmitted internally as a pressure wave. The propagation of this wave is influenced by the physical properties of tissues, with gas-containing organs such as the intestines being particularly susceptible, where the mechanism of injury involves abrupt pressure differentials created at gas-tissue interfaces that can result in tissue disruption, hemorrhage, and, in severe cases, perforation.^13-15^ The connection between injury to the brain and the GI track is well-established (for review see^16^). Brain injuries can result in profound changes in the gut, involving a cascade of changes including disruption of the intestinal barrier, increased infiltration of systemic immune cells, dysmotility, and dysbiosis, enteroendocrine cell dysfunction and disruption in the enteric nervous system and auto nomic nervous system that contribute to negative clinical outcomes following brain injury.^16^

Our prior work in a cohort of SMs (mostly 12B, combat engineers) referred to as breachers, indicated that exposures to blast overpressure may contribute to intestinal permeability (IP), which was likewise associated with deficits in cognitive functioning.^17^ We found evidence of bacterial translocation into circulation following BOP exposures through detection of microbial sequences and quantification of microbial diversity (measured by alpha diversity) that showed a significant stepwise increase.^17^ This observation was further corroborated through the detection of altered levels of IP biomarkers including Zonulin (haptoglobin 2 precursor), Claudin-3, Lipopolysaccharide-Binding Protein (LBP) and Intestinal-Fatty Acid Biding Protein (I-FABP) in the same cohort of SMs.^17^ These IP biomarkers are widely used in clinical and research settings for detection of leaky gut.^18-20^

The present study investigated alterations in IP biomarkers in an independent cohort of SMs with different military occupational specialty (11C, Indirect Fire Infantryman or “mortarmen”), engaged in mortar fire tactical training operations. Physical and psychological symptom data and blood specimens were collected over a period of 6 months from this cohort of mortarmen who participated in 3 tactical training sessions varying in both BOP exposure magnitudes and rates of fire. Motivated by our prior findings in breachers,^17^ we hypothesized that mortarmen exposed to LLB during tactical training would show alterations in levels of IP protein biomarkers (i.e., Zonulin, LBP, Claudin-3, I-FABP). Through exploratory analyses (given limited sample size with small numbers of participating mortarmen), we also aimed to investigate: (1) associations between blast induced changes in these IP biomarkers and participant’s prior history of TBI; and (2) associations between these IP biomarker changes and physical and psychological symptoms using timeseries data collected during tactical training sessions.

## METHODS

### Sample Demographics and Symptom Reporting

Data were collected during 3 separate tactical training evolutions over a 6-month time period at the same military training site. Twenty-two male subjects participated in mortar fire training, involving both 81mm mortar and 120mm mortar and all charge levels, 0-4. During the course of these training sessions, individual blast exposures were measured with wearable pressure sensors (BlackBox Biometrics Blast Gauge, Jessup, MD) and quantified as cumulative impulse (measured in pounds per square inch [psi] × milliseconds [ms]) across the training day.^6,21^ An additional 9 SMs who were also mortarmen from the same unit but did not engage in these training operations were included and for reference heretofore are referred to as unexposed study controls. Blood sample specimens were available for 31 participants investigated in the present study with some participants present for more than 1 study evolution. All participants met military physical requirement standards and were fit for duty. Although the training protocol was open to participation of both male and female SMs, no females participated during the data collection for this study. Participants’ self-reported history of mild traumatic brain injury (mTBI) and approximate self-reported number of career blast exposure events were recorded before engagement in training and are shown in Table 1 and range from 4 to 100k+ self-reported blast exposure events. Blood samples were collected on the training day serially at pre-blast (morning 7:30 AM to 9:00 AM), post-blast (afternoon 4:30 PM to 5:30 PM) on the training day, and follow-up the next day (morning 7:30 AM to 9:00 AM) and stored in −80 °C for downstream experiments. On average within each training session, the post-blast time point corresponded to 1 hour, and follow-up to 16 hour following exposures to blast (an overnight period that likely includes sleep).

Additionally, during each tactical training session, participants completed a 32-item, paper-and-pencil neurotrauma symptom inventory at pre-, post-, and follow-up blast exposure timepoints in conjunction with each blood draw. Items on the symptom survey were the same as that of the Rivermead Post Concussion Symptoms Questionnaire,^22,23^ with additional items included to capture effects previously observed as reported in BOP exposure environments (e.g., tinnitus). These items are consistent with concussion symptomology present in current clinical and research findings.^4,24^ Items on this survey are on a 5-point Likert scale (0 “not experienced at all,” 1 “no more of a problem than before training,” 2 “mild problem–present but don’t really notice and doesn’t concern me,” 3 “moderate problem–I can continue what I am doing but I notice the problem,” 4 “severe problem–constantly present, feels like it could affect my performance”).

### Intestinal Permeability Biomarkers Assayed via Enzyme-Linked Immunosorbent Assay

IP biomarkers including Zonulin, LBP (LPS binding protein), Claudin-3, and I-FABP, were assayed as previously described.^17^ Briefly, plasma levels of Zonulin, LBP, and Claudin-3 were quantified using enzyme-linked immunosorbent assay (ELISA) kits from MyBiosorce (catalog # MBS706368, MBS2024051, and MBS2023694, respectively) and I-FABP levels measured using the ELISA kit from R&D Systems (catalog # DFBP20), following the manufacturer’s protocols for all procedures. All IP biomarkers were measured in duplicate, and the average concentrations were calculated for use in downstream analyses.

### Data and Statistical Analyses

Statistical analyses were performed in R 4.4.0. Group differences for age, service duration, mTBI history and total blast exposure between exposed and unexposed study controls were tested using t-test or Chi-square test. We compared changes in biomarker levels pre versus post/follow-up between the 2 groups (i.e., exposed and unexposed study control participants) for each session. Because of the small sample size for the unexposed study control group, effect sizes measured by Cohen’s d were calculated and reported, instead of testing for significance. Within the exposed group of participants, we investigated whether IP biomarker changes were correlated with magnitude of blast exposures in each training session, with correlations reported as effect sizes measured by Cohen’s d using correlation to effect size conversion via R package effectsize.^25^ To determine the cumulative impact of blast magnitude on IP biomarker changes across sessions, we used a linear mixed effect model as follows via R package lme4 and emmeans:

IPBiomarkerchange=BlastMagnitude+Session+(1∣Subject)

In exploratory analyses we investigated how IP biomarker changes associate with prior mTBI history, or physical/psychological symptoms following blast in line with our previous works.^17,26^ For both of these analyses we used a similar model and R package where the Group variable corresponded to either (a) prior mTBI history (Yes or No) or (b) dichotomized symptom(s) (denoted as 1 if a given symptom was increasing or 0 if not, following blast exposure):

IPBiomarkerchange=Group+Session+(1∣Subject)

Because of the small sample sizes across these comparison groups, differences were reported as effect sizes only, measured by Cohen’s d.

## RESULTS

Of the 31 SMs included in the present study, 22 participants engaged in mortar fire tactical training with an average age of 25.0 ± 3.9 years. All participants engaged in training were serving as mortarmen as their military occupational specialty (MOS 11C). Participants’ self-reported history of mTBI and number of blast exposures were recorded before engagement in training and are described in Table 1. Data and biospecimens were collected over 3 training sessions at the same military site within a 6-month period, with first and second sessions being 4 months apart and the second and third sessions 1 month apart. A total of 50% of participants engaged in 2 or more training sessions and 18% in all 3 training sessions (see Table S1 for participants’ demographics for each session). The average blast exposure magnitudes were 238.0, 556.5, and 98.3 psi*ms for sessions 1, 2, and 3, respectively, where, comparatively, session 3 represented the lowest average magnitude and, correspondingly, had fewest complaints from participating SMs following blast exposure as indicated by changes in symptom reporting at pre versus post/follow-up timepoints (Figure S1).

For each training session, IP biomarker changes were examined at pre versus post/follow up timepoints. We first compared changes in biomarker levels pre versus post/follow-up between the 2 groups, that is, exposed and unexposed study control participants (Figure S2). Secondly, within the exposed group of participants we investigated whether IP biomarker changes were associated with magnitude of blast exposures. Correlations between IP biomarker changes and magnitude of blast exposures in each training session were determined and are reported as effect sizes measured by Cohen’s d (see Table S2). Note that data from session 3 were not included in primary analyses given the low blast exposure magnitudes observed for this session; however, session 3 results are provided in the supplemental materials (Figure S3 and Table S2).

**Figure 1.**
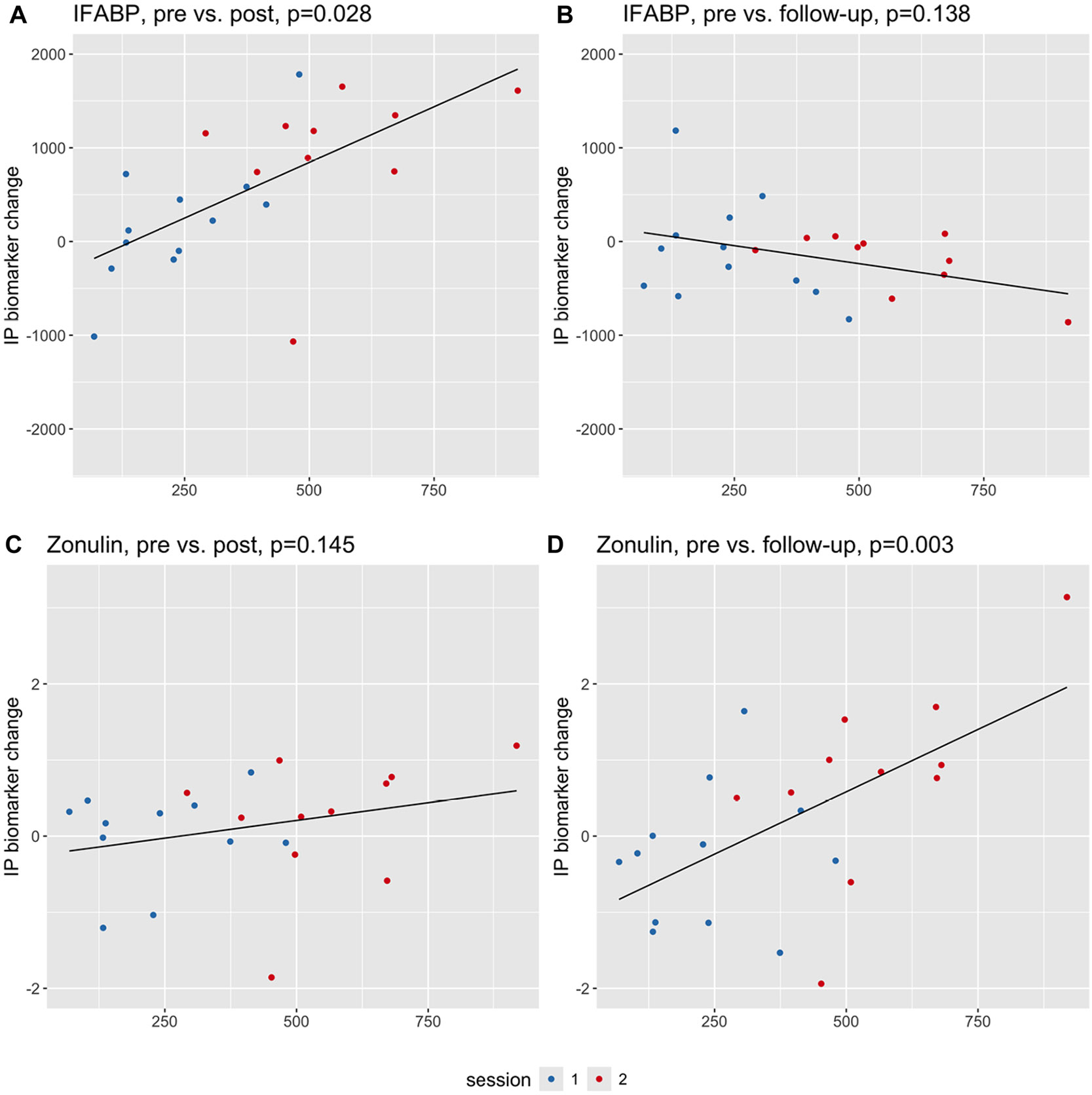
Blast exposure confers changes in intestinal permeability biomarker levels. Scatterplots show associations between blast exposure magnitude and intestinal permeability biomarker changes for pre versus post/follow-up timepoints including: (A) intestinal-fatty acid binding protein pre versus post and (B) intestinal-fatty acid binding protein pre versus follow-up measured in pg/ml; and (C) Zonulin pre versus post and (D) Zonulin pre versus follow-up measured in ng/ml. Data shown correspond to sessions 1 and 2, with P-values for association between blast exposure magnitude and cumulative changes in intestinal-fatty acid binding protein and Zonulin across both sessions provided in each panel.

**Figure 2.**
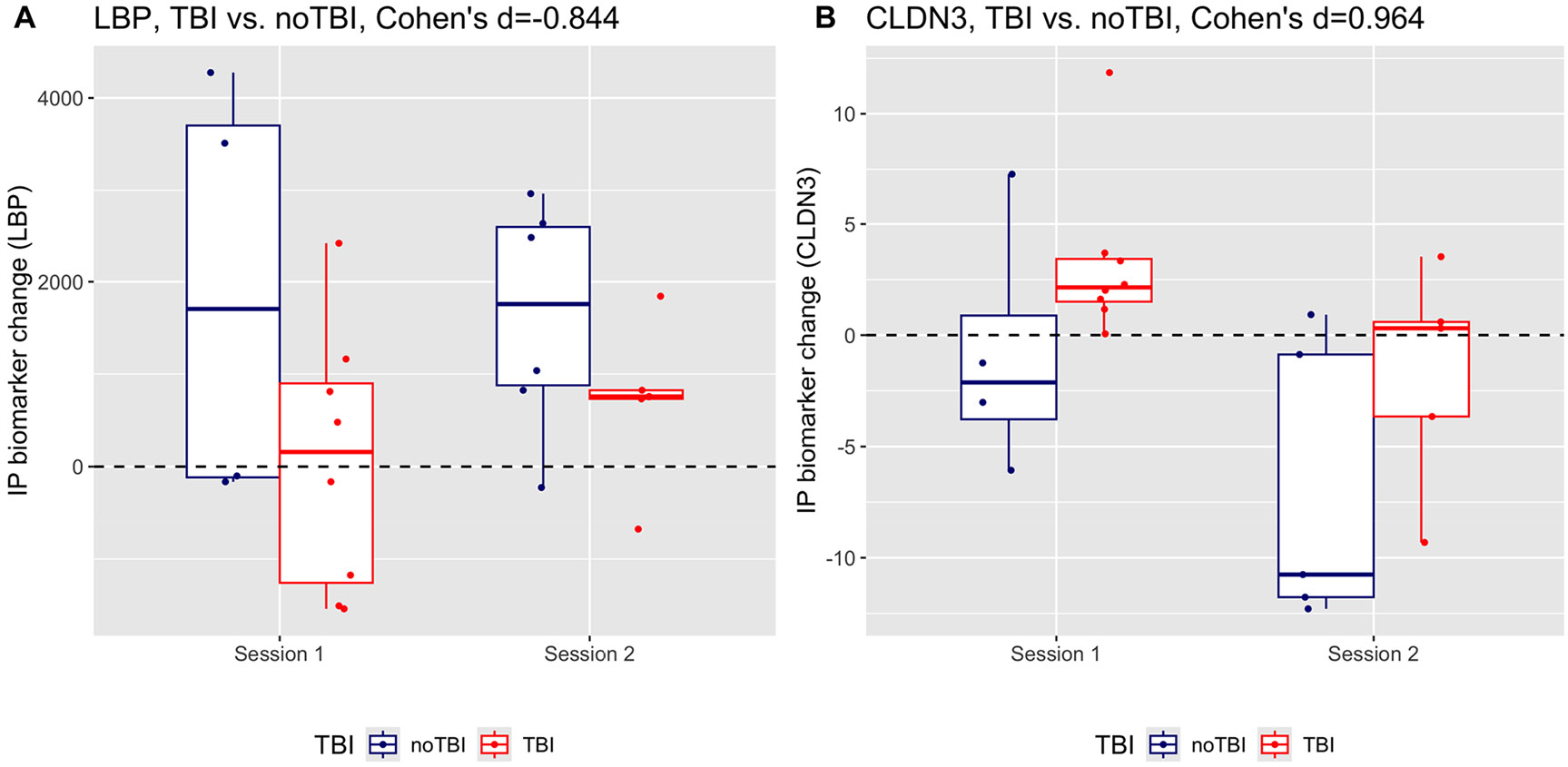
Blast-associated intestinal permeability biomarker responses differ by mild traumatic brain injury history. Intestinal permeability biomarker changes cumulatively between pre and post time points across mortar fire training sessions 1 and 2 are shown for (A) Lipopolysaccharide-Binding Protein, measured in ng/ml, and (B) Claudin-3, measured in ng/ml separately in those with or without prior history of mild traumatic brain injury. Only group differences with large (∣d∣>0.8) effect size are shown.

**Figure 3.**
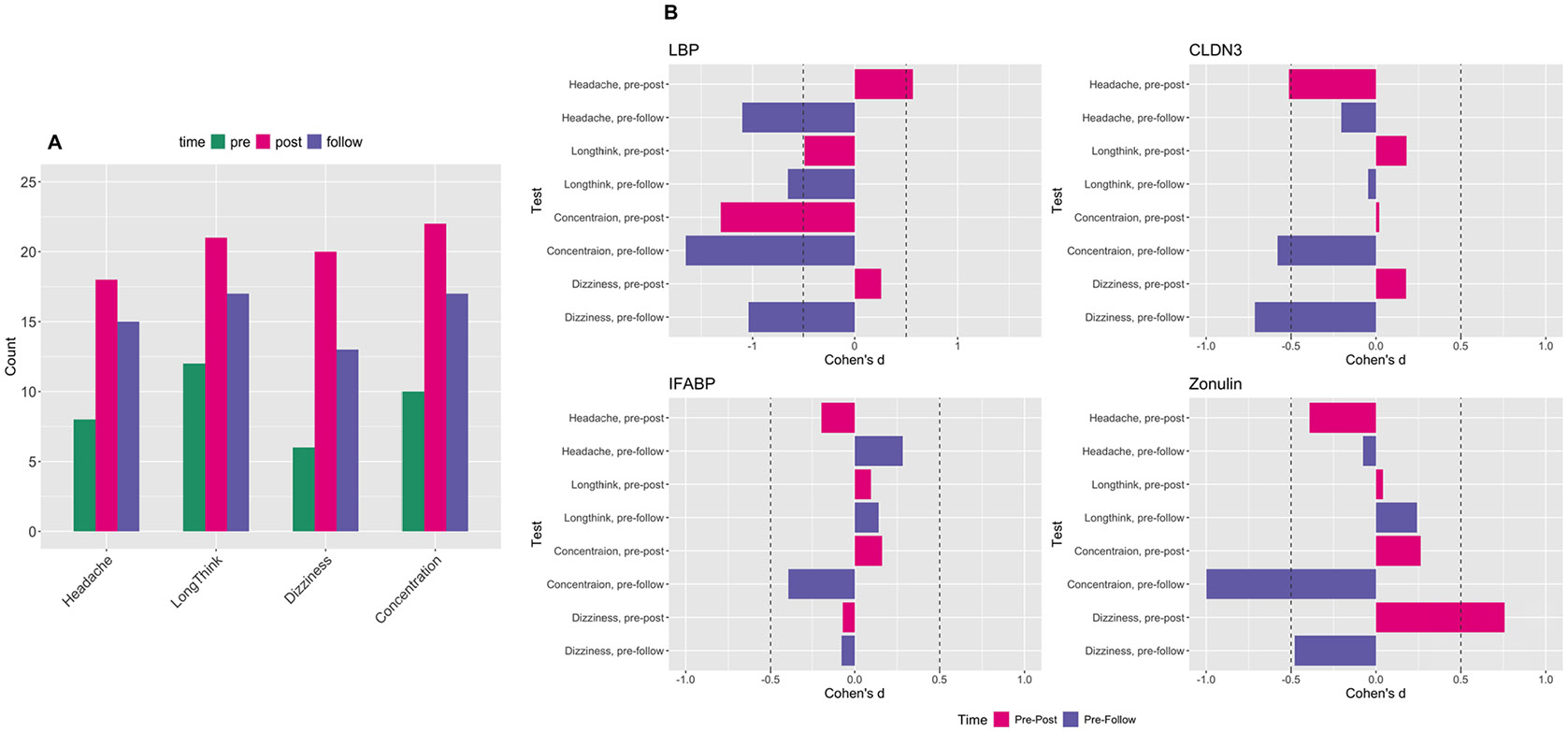
Intestinal permeability biomarker changes associate with neurotrauma symptoms following blast. Bar plots show (A) most frequently reported neurotrauma symptoms reported at pre, post, and follow-up timepoints after blast and (B) effect sizes for associations between cumulative changes in intestinal permeability Biomarkers and neurotrauma symptoms at pre versus post/follow-up reported by Cohen’s d: negligible (∣d∣<0.2), small (0.2≤∣d∣<0.5), medium (0.5≤∣d∣<0.8), and large effect size beyond.

Furthermore, associations of IP biomarkers with blast magnitude cumulatively across the first 2 training sessions using a linear mixed-effects model identified significant associations for I-FABP (pre vs. post timepoints, *P* = .028, *t* = 2.371), and Zonulin (pre vs. follow-up timepoints, *P* = .003, *t* = 3.390; Figure 1). Associations for LBP and CLND3 IP biomarkers were not significant (see Figure S4). We also detected associations between TBI history and blast induced LBP and CLDN3 alterations, with large effect sizes (Cohen’s *d* = −0.844 and 0.964 for LBP and CLDN3 IP biomarkers, respectively; see Figure 2). Associations between IP biomarker changes and neurotrauma symptom reporting including increased headaches, difficulty with concentration, taking longer to think, and dizziness (symptoms most frequently reported during tactical training) were also investigated (see Figure 3 for most frequently reported symptoms in session 1 and 2, and Figure S1 for neurotrauma symptoms reported across all 3 sessions). Moderate to large marginal effect sizes were also observed for associations between cumulative changes in LBP and CLDN3 and neurotrauma symptoms across sessions 1 and 2 at pre versus post/follow-up timepoints (Figure 3).

## DISCUSSION

This study corroborates our prior findings in breachers^17^ in an independent cohort of SMs engaged in mortar fire training. In the present study, we show alterations in levels of IP protein biomarkers that indicate blast exposure contributes to gut permeability, which associates with reported neurotrauma symptoms following blast exposures. In line with prior findings,^26^ we also found that blast-associated IP biomarker changes were significantly correlated with prior mTBI history. In sum, the present study contributes to an emerging body of evidence indicating that LLB contributes to gut permeability.

For LBP and CLDN3, the direction of change in IP biomarkers and neurotrauma symptoms following exposures to blast in these mortarmen were consistent with our prior observations in breachers^17^ (see Figure 3 for mortarmen, Figure S5 for breachers reported previously^17^). Also, across both cohorts, increases in I-FABP and Zonulin IP biomarkers were observed after blast exposure, though the dynamics in timing of the biomarker response varied between the mortarmen and the breachers. In contrast to the breacher cohort, the mortarmen in the present study were younger (average age 25.0 ± 3.7, as compared to breachers with average age of 30.0 ± 6.3, *t* = 3.76, *P* < .001). The mortarmen had a shorter duration in military service (4.8 ± 3.1) versus the breachers (8.9 ± 4.6, *t* = 4.00, *P* < .001). Also, by virtue of their MOS, the average total blast exposure for the mortarmen was significantly greater consisting of 66,453 ± 137,445 and for the breachers 2,819 ± 11,704 (*t* = 2.48, *P* = .019) with the majority of the participants in both cohorts endorsing prior history of mTBI (64% and 57% for mortarmen and breachers, respectively). Despite differences in the cohorts’ demographic and exposure histories, the military occupational specialties of the participants investigated in these studies represent environments in which they are exposed to high levels of peak overpressure. Consequently, contributing to the observed findings related to alterations in IP biomarker response across both cohorts. Both cohorts also exhibit a decrease in cognitive functioning after blast exposure, which also associated with changes in IP biomarker levels. Overall, findings from this and our prior work^17^ in SMs with LLB exposures show, in real-world military operational settings, that exposures to blast can contribute to gut permeability, and that prior exposures or injuries such as mTBI history, may influence differential IP biomarker response following blast.

Studies in military populations provide a unique opportunity for advancing our understanding of the brain-gut axis in context of physical and psychological stressors, in real-world settings, during tactical training and combat. Our own work in breachers and mortarmen showing the potential impact of blast exposures on the intestinal barrier is further supported by other findings from studies of SMs exposed to physical and psychological stressors during military operations. In particular, studies investigating physical and psychological stressors during a 6-week combat training course comprised of combat simulation and high-intensity medical evacuation exercises found increases in symptoms of stress, anxiety, and depression, with concomitant increases in IP and inflammation,^27^ as well as changes in metabolic responses.^28^ A study of soldiers involved in a 4-day arctic training exercise who endured high physical exertion also found increases in IP and inflammation, as well as changes in gut microbiota and metabolite composition.^29^ Finally, a study involving Marines engaged in a 7.5 day Survival, Evasion, Resistance and Escape training, specifically found alterations in the LBP IP biomarker following training that was associated with increased mood disturbances and symptoms of depression, but not with changes in cognitive performance, in contrast to our findings with blast exposures presented herein and previously.^30^ There is a growing recognition of the impact of military relevant stressors on gut health, especially since personnel often operate in extreme environments with disrupted sleep, exposure to pathogens and toxins, and excessive noise, as well as extreme stress and strenuous physical exertion, that impact their physical and psychological health.^31^ These exposures have shown to be related to such chronic symptoms as gastroenteritis, insomnia, substance use disorders, bipolar disorders, and severe depression in Veterans that has also been shown to be associated with distinct gut microbiome signatures.^32^

This study has several limitations. As an observational human study conducted within military operational settings, clinician-administered assessments of neurotrauma symptoms were not performed since it was not feasible and would have interfered with the objectives of the training evolutions. Additionally, history and mechanism of mTBI were both based on self-report, with no data collected on the date of injury. No data on participants’ diets were collected, since the initial study protocol used to collect these samples was developed with the focus to assess the effects of blast on cognitive performance and self-reported neurotrauma symptoms; however, the majority of these trainings occurred in remote locations and food intake was likely different from a normal diet and could have included Meals Ready to Eat (MRE). Persistent intake (i.e., 21 straight days) of only MRE has been shown to alter the gut microbiome without altering IP or gastrointestinal symptoms.^33^ Findings from this work may motivate future Department of Defense (DoD) and military operational data collection efforts to further investigate the relationship between blast exposure and gut health, where data on the participants’ diets and other relevant GI conditions can be collected. Future operational data collection efforts may also serve to investigate the blast exposure-gut health relationship using alternate measures of individual exposure besides the wearable sensors reported here. These may include estimates of individual exposures based on observation monitoring using, for example, the Blast Overpressure (BOP) Tool,^34^ within the Range Manager Toolkit. Such tools offer the capabilities to monitor and to investigate blast exposure of SMs in line with the interim DoD guidance for managing and documenting brain health risk from blast overpressure, which can also be entered as part of individual exposure records of SMs for use in future IP biomarker studies. Furthermore, though this study included unexposed study controls who did not engage in mortar fire trainings, the activities of these participants were not controlled nor tracked. It is possible that these individuals may have engaged in activities that could have impacted study measures of interest. Since the training protocol requires multiple days of military training exercises, it is challenging to identify available SMs to serve as controls. As such, this study involving mortarmen and our prior work in breachers^17^ exposed to LLB is principally based on a longitudinal design with repeated measures, where each participant’s pre-exposure is their own control when comparing symptom and biomarker measures pre versus post/follow-up blast exposure. This study also lacks representation of both sexes. Even though the training protocol was open to both sexes, there were no females in this training cohort. As an observational study, data is collected where blast exposure occurs in existing operational training environments, and presently represents the bias in predominantly male SMs that participate in these training protocols. There was not a sufficiently sized sample to investigate longer-term longitudinal data on both bacterial species and IP measures following blast exposures, which were not feasible given the scope of the parent protocol under which the symptom and biospecimen data were collected, as well as the participants’ duties although in military service. Finally, it is possible that the ELISA assays used for quantification of IP biomarkers were not sensitive enough to detect subtle changes in IP biomarkers, which can be investigated in future studies using other approaches as digital ELISA (or single molecule enzyme-linked immunosorbent assays).

In conclusion, our study findings underscore the importance of the role of blast exposure in gut permeability and of investigating the role of the gut-brain axis in the context of military exercises. Exposures to blast and gut permeability evidenced by bacterial translocation and IP demonstrated by the present study suggest a highly promising treatment target for our SMs and Veterans; thereby highlighting the importance of identifying new potential preventive and post-facto treatments that target the gut in those exposed during military tactical operations. Should links between repeated low-level blast exposure and disturbed gut health be supported through studies with large numbers of participating SMs and Veterans, this would be transformative in contributing towards the development of novel treatment strategies aimed at improving the gut microbiome as a means of mitigating the long-term health impacts of such exposures. Although many factors may influence the gut microbiome, future directions of this research may focus on diet, as this appears to play a particularly important role in influencing its composition and associated metabolites,^35^ which, along with the non-invasive nature of dietary interventions, makes it a particularly promising avenue for sculpting the gut microbiota to enhance gut health amongst SMs with repeated exposures to blast as part of their military occupational specialty.

## Supplementary Material

Supplementary Material

SUPPLEMENTARY MATERIAL

Supplementary material is available at *Military Medicine* online.

## Figures and Tables

**Table 1.** Demographics, Military Occupation, and Exposure History

Group	Exposed	Unexposed	Test and *P*-value
*N*	22	9	
Age (years)	25.0 ± 3.9	25.0 ± 3.4	*t*-test, *P* = .98
Service duration (years)	5.0 ± 3.5	4.4 ± 1.6	*t*-test, *P* = .55
mTBI	14 (64%)	5 (56%)	*χ*^2^ test, *P* = .99
MOS	11C Mortarman: 22 (100%)	11C Mortarman: 5 (56%) Other: 4 (44%)	*χ*^2^ test, *P* = .01
Total blast exposure	1-1,000: 5 1,001-10,000: 6 10,001-30,000: 5 30,001-100,000: 2 100,000+: 2 Missing: 2	1-1,000: 4 1,001-10,000: 2 10,001-30,000: 0 30,001-100,000: 1 100,000+: 2	*χ*^2^ test, *P* = .43

Data shown represent SMs’ age, service years in the military, history of mTBI, MOS, and blast exposure history categorized within pre-defined exposure ranges. Herein unexposed refers to the SMs in the study control group, who did not engage in mortar fire trainings.

Abbreviations: MOS, military occupational specialty; mTBI, mild traumatic brain injury; SMs, Service Members.

## Data Availability

The datasets presented in this article are not readily available because such information as the site location, data collection date, date of birth, and gender, may reasonably allow identification of participants in the study. Deidentified raw data (not including these variables) supporting the conclusions of this article can be made available by the authors, on condition that institutional and ethical requirements for sharing data are met.
