# Supplementary material for: Associations Between Blast Exposures and Intestinal Permeability and Neurotrauma Symptoms During Mortar Fire Military Tactical Training Operations
Source: Mil Med. Author manuscript; Available in PMC 2026 Jun 23. (PMC13288333; doi:10.1093/milmed/usaf478)
Supplement: Supplementary Material [file NIHMS2140295-supplement-Supplementary_Material.docx]

Table S1: Demographics, military occupation, and exposure history shown separately for each training session

|  | Group | N | TBI (%) | Age | Service duration |
| --- | --- | --- | --- | --- | --- |
| Session 1 | Not exposed | 5 | 0(0%) | 26.6±3.36 | 5±1.87 |
|  | Exposed | 12 | 8(67%) | 25±2.95 | 3.93±1.95 |
|  |  |  |  |  |  |
| Session 2 | Not exposed | 5 | 1(20%) | 26.4±3.44 | 4.2±0.84 |
|  | Exposed | 11 | 5(45%) | 24.5±3.47 | 4±2.14 |
|  |  |  |  |  |  |
| Session 3 | Not exposed | 5 | 2(40%) | 26.4±3.44 | 4.2±0.84 |
|  | Exposed | 14 | 9(64%) | 25.5±4.34 | 5.93±4.10 |

Data shown represent SMs’ history of mTBI, age, and service years in the military separated by session. Herein unexposed refers to the SMs in the study control group, who did not engage in mortar fire trainings.

Figure S1: Symptom reporting during tactical training


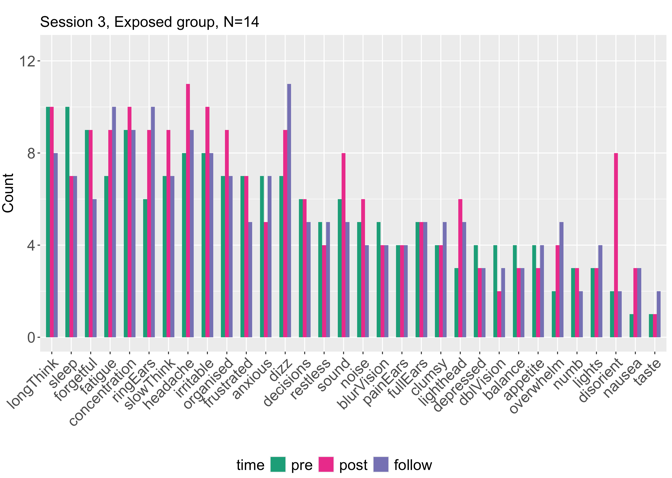

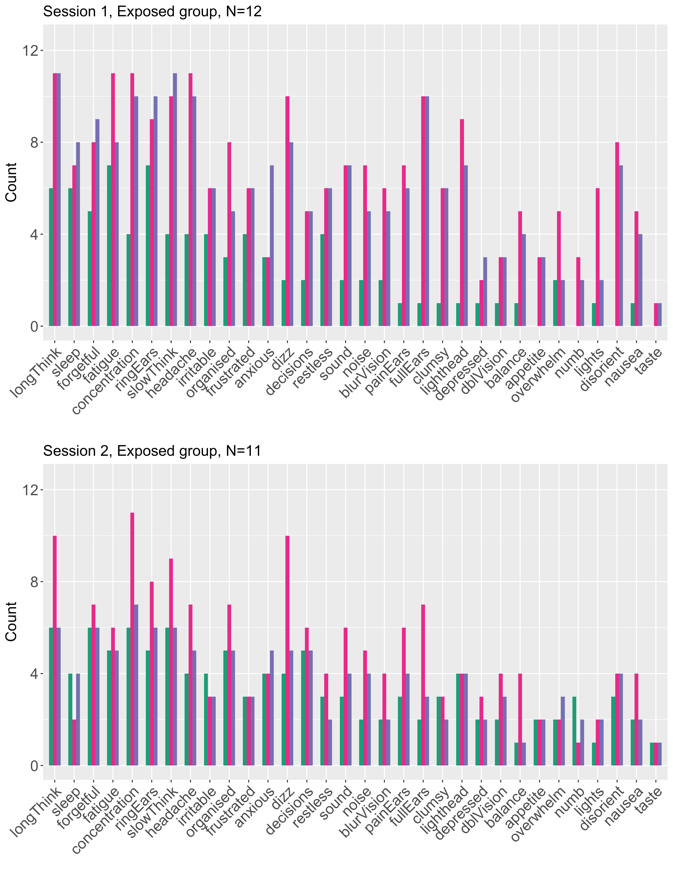


Bar plots show symptoms reported at pre, post, and follow-up timepoints after exposures to LLB across the 3 sessions, corresponding to (A) session 1, (B) session 2, and (C) session 3.

**Figure S2: Difference in IP Biomarker Levels in Exposed vs. Unexposed SMs**
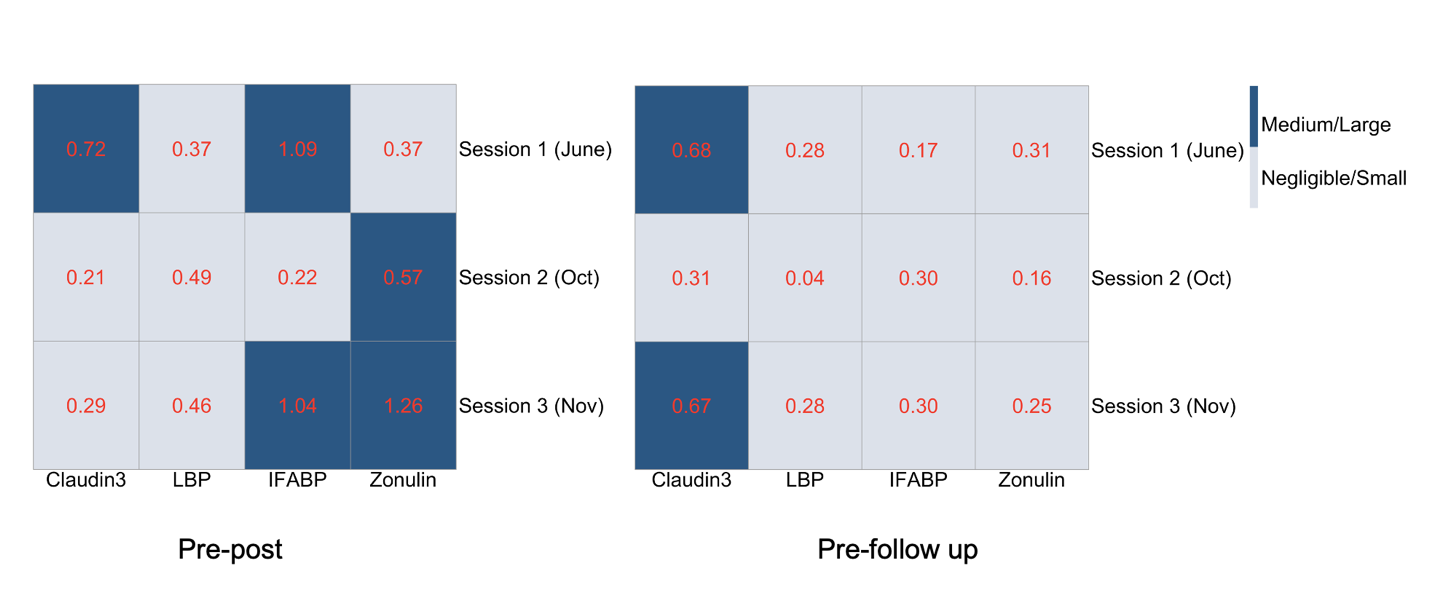


Heatmap shows magnitude of differences in IP biomarker levels in exposed vs. unexposed SMs across the three training sessions for (A) pre-post and (B) pre-follow up timepoints measured by Cohen’s d: negligible (|d|<0.2), small (0.2≤|d|<0.5), medium (0.5≤|d|<0.8), and large effect size beyond.

Table S2: Effect size of correlations between IP Biomarker Changes and Blast Magnitude

|  | Pre vs. Post | | | Pre vs. Follow-up | | |
| --- | --- | --- | --- | --- | --- | --- |
| IP Biomarker | Session 1  Cohen’s d (Magnitude) | Session 2  Cohen’s d (Magnitude) | Session 3  Cohen’s d (Magnitude) | Session 1  Cohen’s d (Magnitude) | Session 2  Cohen’s d (Magnitude) | Session 3  Cohen’s d (Magnitude) |
| CLDN3 | 0.402  (Small) | -0.092  (Negligible) | -0.285  (Small) | -0.579  (Medium) | -0.084  (Negligible) | -0.799  (Medium) |
| LBP | -0.912  (Large) | -0.570  (Medium) | 1.757  (Large) | -1.875  (Large) | -0.505  (Medium) | 1.019  (Large) |
| IFABP | 2.174  (Large) | 0.673  (Medium) | -0.374  (Small) | -0.807  (Large) | -0.244  (Small) | 0.016  (Negligible) |
| Zonulin | 0.232  (Small) | 0.657  (Medium) | -0.440  (Small) | 0.335  (Small) | 1.589  (Large) | -0.966  (Large) |

Correlations between pre vs. post/follow up IP biomarker changes and exposure magnitude across all sessions were converted from Spearman correlations to effect sizes measured via Cohen’s d.

**Figure S3: Blast Exposure Confers IP Biomarker Change shown across all sessions**


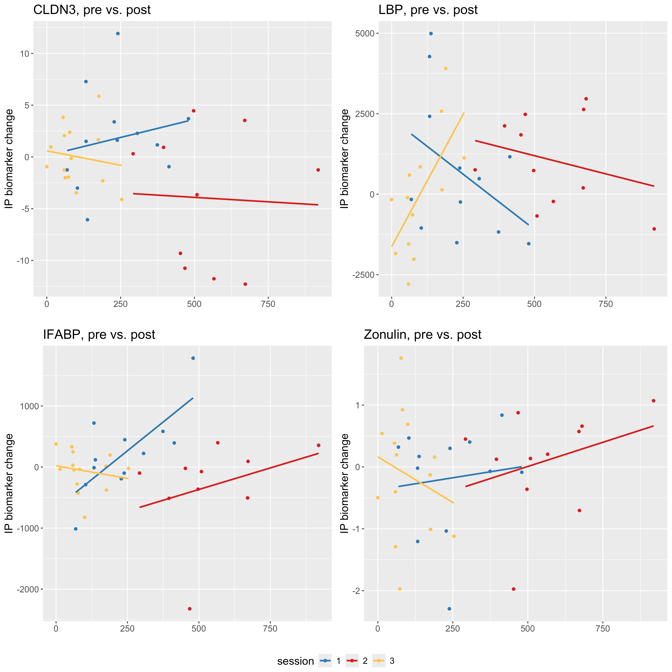

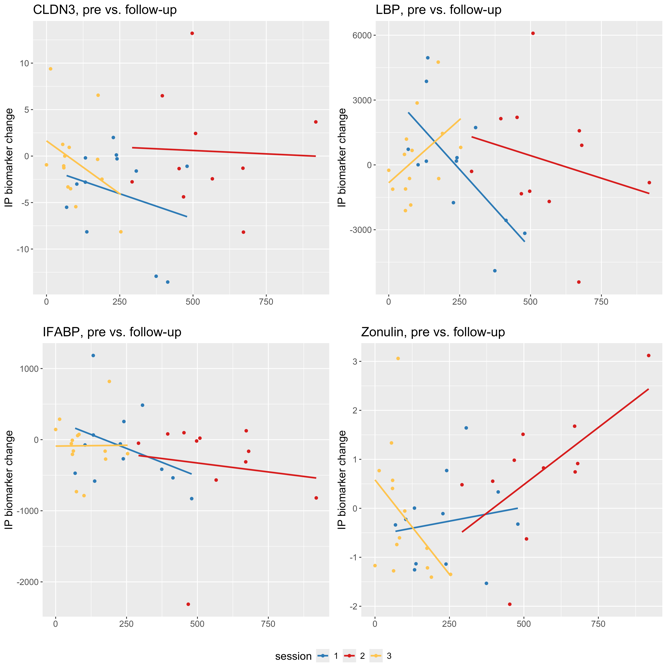


Scatter plots depict associations between blast exposure magnitude and changes in IP biomarkers pre vs. post/follow-up for all IP biomarkers examined across all sessions with session 1 depicted in blue, session 2 depicted in red and session 3 depicted in yellow. Effect sizes for these associations are provided in Table S2.

**Figure S4: Blast Exposure Confers change in IP Biomarker levels**


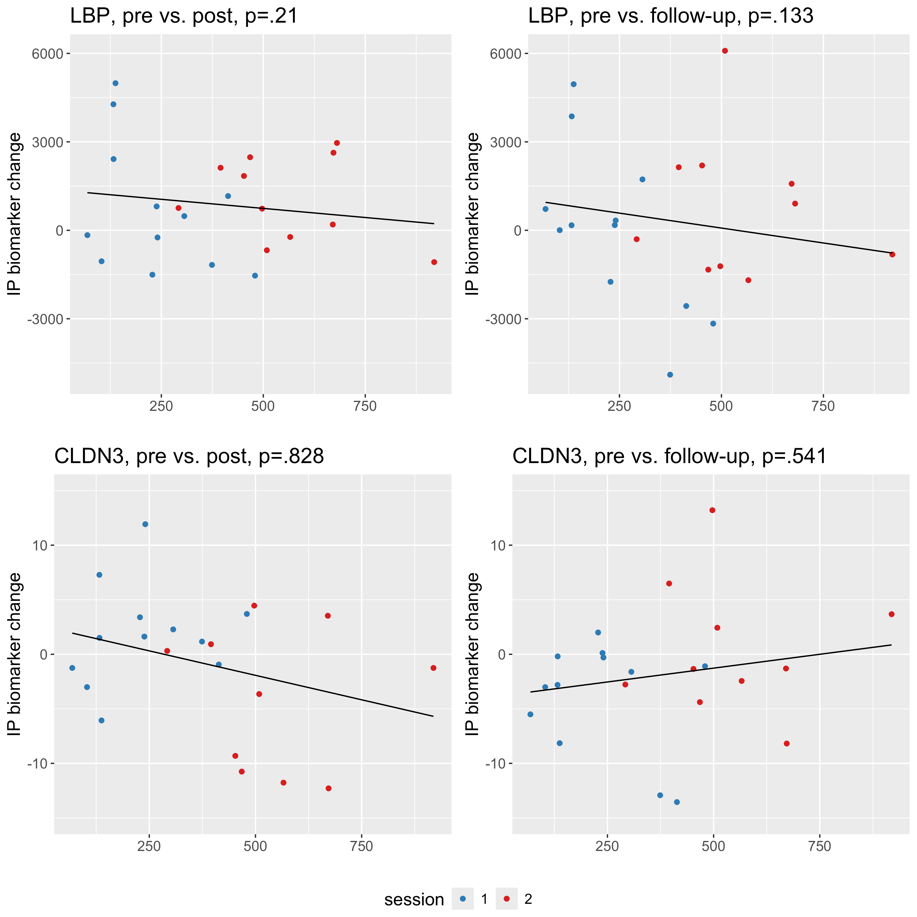


Scatter plots show associations between blast exposure magnitude and IP biomarker changes for pre vs. post/follow-up timepoints including: (A) LBP pre vs. post and (B) LBP pre vs. follow-up, both measured in pg/ml; and (C) CLDN3 pre vs. post and (D) CLDN3 pre vs. follow-up, both measured in ng/ml. Data shown correspond to sessions 1 (depicted in blue) and 2, (depicted in red). With p-values for association between blast exposure magnitude and cumilitive changes in LBP and CLDN3 across both sessions provided in each panel.

**Figure S5: IP Biomarker Changes Associate with Neurotrauma Symptoms Following Blast in Breachers**


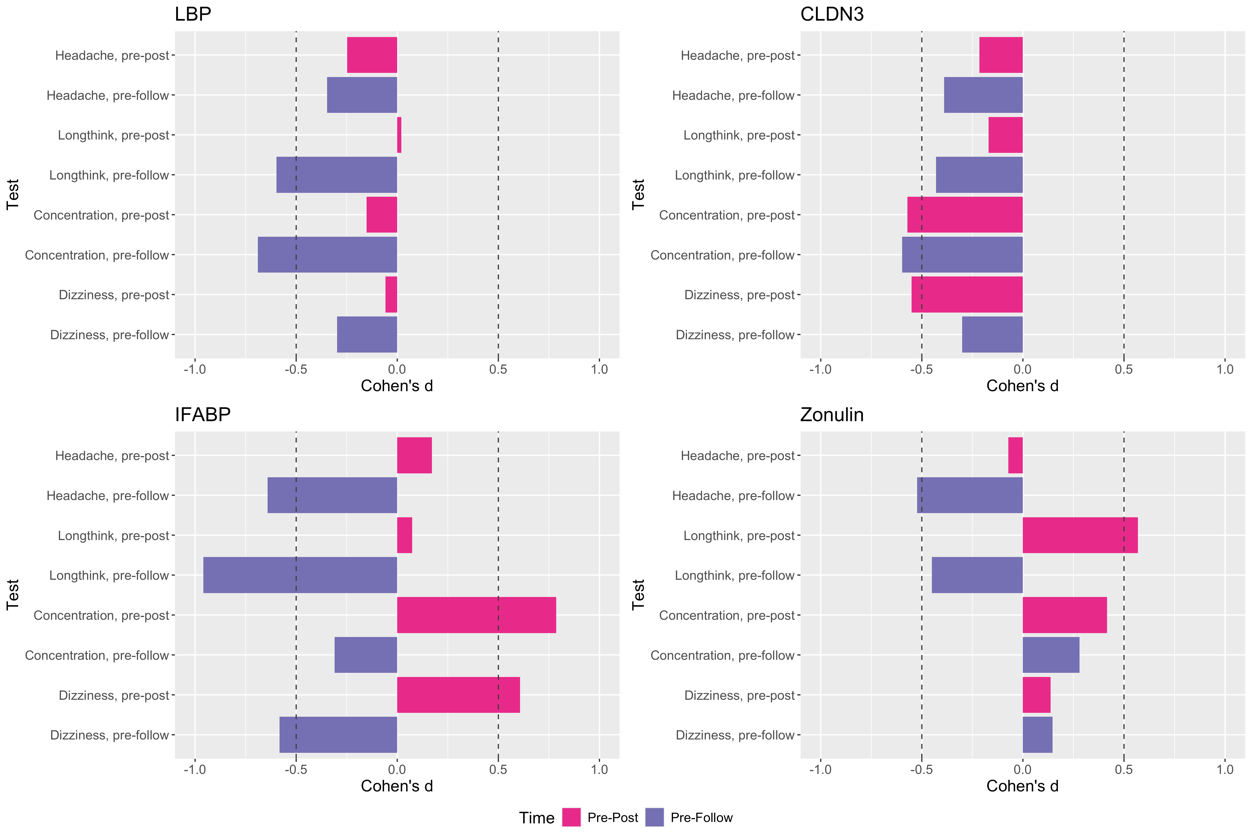


Bar plots show effect sizes for associations between cumilitive changes in LBP and CLDN3 and neurotrauma symptoms at pre vs. post/follow-up reported by Cohen’s d: negligible (|d|<0.2), small (0.2≤|d|<0.5), medium (0.5≤|d|<0.8), and large effect size beyond. Data reported previously [14] in breachers engaged in tactical training.
